# Tracking Genomic Cancer Evolution for Precision Medicine: The Lung TRACERx Study

**DOI:** 10.1371/journal.pbio.1001906

**Published:** 2014-07-08

**Authors:** Mariam Jamal-Hanjani, Alan Hackshaw, Yenting Ngai, Jacqueline Shaw, Caroline Dive, Sergio Quezada, Gary Middleton, Elza de Bruin, John Le Quesne, Seema Shafi, Mary Falzon, Stuart Horswell, Fiona Blackhall, Iftekhar Khan, Sam Janes, Marianne Nicolson, David Lawrence, Martin Forster, Dean Fennell, Siow-Ming Lee, Jason Lester, Keith Kerr, Salli Muller, Natasha Iles, Sean Smith, Nirupa Murugaesu, Richard Mitter, Max Salm, Aengus Stuart, Nik Matthews, Haydn Adams, Tanya Ahmad, Richard Attanoos, Jonathan Bennett, Nicolai Juul Birkbak, Richard Booton, Ged Brady, Keith Buchan, Arrigo Capitano, Mahendran Chetty, Mark Cobbold, Philip Crosbie, Helen Davies, Alan Denison, Madhav Djearman, Jacki Goldman, Tom Haswell, Leena Joseph, Malgorzata Kornaszewska, Matthew Krebs, Gerald Langman, Mairead MacKenzie, Joy Millar, Bruno Morgan, Babu Naidu, Daisuke Nonaka, Karl Peggs, Catrin Pritchard, Hardy Remmen, Andrew Rowan, Rajesh Shah, Elaine Smith, Yvonne Summers, Magali Taylor, Selvaraju Veeriah, David Waller, Ben Wilcox, Maggie Wilcox, Ian Woolhouse, Nicholas McGranahan, Charles Swanton

**Affiliations:** 1 Translational Cancer Therapeutics Laboratory, University College London Cancer Institute, London, United Kingdom; 2 Department of Medical Oncology, University College London Hospitals, London, United Kingdom; 3 Cancer Research UK & UCL Cancer Trials Centre, London, United Kingdom; 4 Cancer Studies and Molecular Medicine, University of Leicester, Leicester, United Kingdom; 5 Cancer Research UK Manchester Institute, Manchester, United Kingdom; 6 Immune Regulation and Tumour Immunotherapy Laboratory, University College London Cancer Institute, London, United Kingdom; 7 Department of Medical Oncology, Birmingham Heartlands Hospital, Birmingham, United Kingdom; 8 Department of Pathology, University College London Hospitals, London, United Kingdom; 9 Department of Bioinformatics and BioStatistics, Cancer Research UK, London Research Institute, London, United Kingdom; 10 Institute of Cancer Studies, University of Manchester and The Christie Hospital, Manchester, United Kingdom; 11 Department of Respiratory Medicine, University College London Hospitals, London, United Kingdom; 12 Department of Medical Oncology, Aberdeen University Medical School & Aberdeen Royal Infirmary, Aberdeen, Scotland, United Kingdom; 13 Department of Cardiothoracic Surgery, Heart Hospital, London, United Kingdom; 14 Department of Medical Oncology, University of Leicester & Leicester University Hospitals, Leicester, United Kingdom; 15 Department of Clinical Oncology, Velindre Hospital, Cardiff, Wales, United Kingdom; 16 Department of Pathology, Aberdeen University Medical School & Aberdeen Royal Infirmary, Aberdeen, Scotland, United Kingdom; 17 Department of Pathology, University of Leicester & Leicester University Hospitals, Leicester, United Kingdom; 18 The Advanced Sequencing Facility, London Research Institute, London, United Kingdom; 19 Department of Radiology, University Hospital Llandough, Cardiff, Wales, United Kingdom; 20 Department of Pathology, University Hospital Llandough, Cardiff, Wales, United Kingdom; 21 Department of Respiratory Medicine, University of Leicester & Leicester University Hospitals, Leicester, United Kingdom; 22 Department of Systems Biology, Technical University of Denmark, Kongens Lyngby, Denmark; 23 Department of Respiratory Medicine, University Hospital of South Manchester, Manchester, United Kingdom; 24 Cancer Research UK Manchester Institute, Manchester, United Kingdom; 25 Department of Cardiothoracic Surgery, Aberdeen University Medical School & Aberdeen Royal Infirmary, Aberdeen, United Kingdom; 26 Department of Respiratory Medicine, Aberdeen University Medical School & Aberdeen Royal Infirmary, Aberdeen, United Kingdom; 27 Department of Clinical Immunology, University of Birmingham, Birmingham, B15 2TT; 28 North West Lung Centre, University Hospital of South Manchester, Manchester, United Kingdom; 29 Department of Respiratory Medicine, University Hospital Llandough, Cardiff, Wales, United Kingdom; 30 Aberdeen Biomedical Imaging Centre, University of Aberdeen, Aberdeen, United kingdom; 31 Department of Radiology, Birmingham Heartlands Hospital, Birmingham, United Kingdom; 32 Department of IT, London Research Institute, London, United Kingdom; 33 Independent Cancer Patient's Voice, London, united Kingdom; 34 Department of Pathology, University Hospitals of South Manchester, Manchester; 35 Department of Cardiothoracic Surgery, University Hospital Llandough, Cardiff, Wales, United Kingdom; 36 Department of Cellular Pathology, Birmingham Heartlands Hospital, Birmingham, United Kingdom; 37 Department of Thoracic Surgery, Birmingham Heartlands Hospital, Birmingham, United Kingdom; 38 The Christie Hospital, Manchester, United Kingdom; 39 Department of Biochemistry, University of Leicester, Leicester, United Kingdom; 40 Translational Cancer Therapeutics Laboratory, London Research Institute, London, United Kingdom; 41 Department of Cardiothoracic Surgery, University Hospitals of South Manchester, Manchester, United Kingdom; 42 Department of Radiology, University Hospitals of South Manchester, Manchester, United Kingdom; 43 Department of Medical Oncology, University Hospital of South Manchester, Manchester, United Kingdom; 44 Department of Radiology, University College London Hospitals, London, United Kingdom; 45 Department of Cardiothoracic Surgery, University of Leicester & Leicester University Hospitals, Leicester, United Kingdom; 46 School of Cancer Sciences, University of Birmingham, Birmingham, United Kingdom; 47 Department of Respiratory Medicine, Birmingham University Hospital, Birmingham, United Kingdom

## Abstract

The importance of intratumour genetic and functional heterogeneity is increasingly recognised as a driver of cancer progression and survival outcome. Understanding how tumour clonal heterogeneity impacts upon therapeutic outcome, however, is still an area of unmet clinical and scientific need. TRACERx (TRAcking non-small cell lung Cancer Evolution through therapy [Rx]), a prospective study of patients with primary non-small cell lung cancer (NSCLC), aims to define the evolutionary trajectories of lung cancer in both space and time through multiregion and longitudinal tumour sampling and genetic analysis. By following cancers from diagnosis to relapse, tracking the evolutionary trajectories of tumours in relation to therapeutic interventions, and determining the impact of clonal heterogeneity on clinical outcomes, TRACERx may help to identify novel therapeutic targets for NSCLC and may also serve as a model applicable to other cancer types.

## Abstract 2

TRACERx, a prospective study of patients with primary non-small cell lung cancer, aims to map the genomic landscape of lung cancer by tracking clonal heterogeneity and tumour evolution from diagnosis to relapse.

## Introduction

Each patient's cancer has a unique genomic landscape, often comprised of populations of genetically distinct, separated subclones with the potential to undergo dynamic evolutionary processes throughout the disease course [1],[2]. One of the major challenges in achieving the goal of precision medicine lies in obtaining an accurate view of this genomic landscape, in order to choose the appropriate therapeutic regimen [3]. Intratumour heterogeneity poses a challenge in that a single tumour biopsy may not fully capture the current or future tumour landscape and merely represents a “snapshot” of the disease in space and time. Several studies have demonstrated branched evolution in different tumour types, including breast [4],[5], pancreatic [6], kidney [7], colorectal [8], and prostate [9] cancers, as well as haematological malignancies such as chronic lymphoblastic leukaemia [1] and acute lymphoblastic leukaemia [10]. Understanding how tumour clonal heterogeneity impacts upon clinical outcome, and how cancer subclones compete, adapt, and evolve through the disease course in relation to therapy, is an area of unmet clinical and scientific need. Lung TRACERx (TRAcking non-small cell lung Cancer Evolution through therapy [Rx], ClinicalTrials.gov number, NCT01888601), is a prospective study in primary non-small cell lung cancer (NSCLC), which through multiregion and longitudinal tumour sampling and sequencing, aims to define the genomic landscape of NSCLC and to understand the impact of tumour clonal heterogeneity upon therapeutic and survival outcome.

## Overview of Lung TRACERx

**Figure 1:**
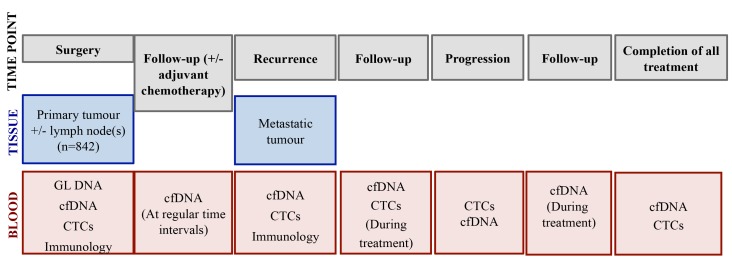
Sample collection in TRACERx.

**Figure 2:**
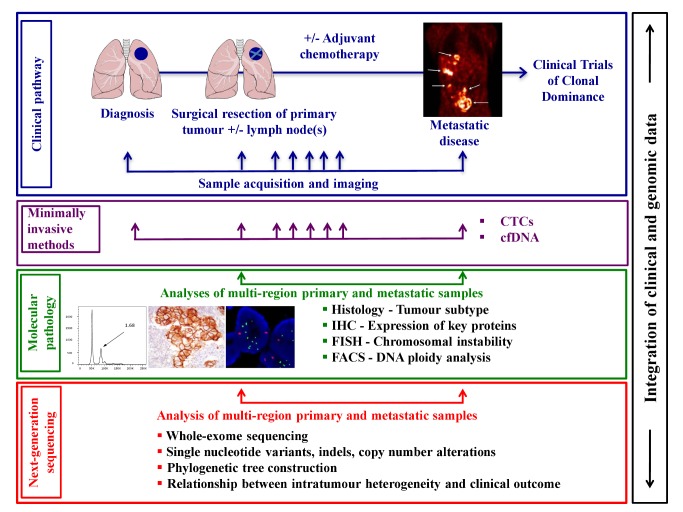
Schematic of an integrated clinical approach to understanding the impact of intratumour heterogeneity upon disease progression and clinical outcome. Abbreviations: cfDNA, circulating-free tumour DNA; CTCs, circulating tumour cells; FACS, fluorescence-activated cell sorting; FISH, fluorescence in situ hybridisation; IHC, immunohistochemistry. Lungs diagram adapted from “Lungs diagram simple” from Patrick J. Lynch, Wikimedia Commons under CY-BY 2.5. Metastatic disease image from Haubner, et al. (2005) PLoS Med 2: e70. doi:10.1371/journal.pmed.0020070. Images of FACS analysis, immunohistochemistry, and FISH obtained from the Swanton lab.

Lung TRACERx incorporates longitudinal sample collection from diagnosis to relapse in order to investigate how each cancer responds to treatment, the potential mutational processes and mechanisms involved in drug resistance, and development of metastatic disease. Although here we discuss TRACERx in NSCLC, the proposed longitudinal sample collection and study template is also relevant to other tumour types. TRACERx, conducted across six sites in the United Kingdom (London, Leicester, Manchester, Aberdeen, Birmingham, and Cardiff), will enrol 842 patients with primary NSCLC stages I-IIIA over an accrual period of four years with a total five-year follow-up per patient. Primary surgically resected NSCLC tumours and associated lymph nodes, surplus to diagnostic requirements, will be subject to multiregion sampling and subsequent whole-exome and/or whole-genome sequencing. In patients suffering disease recurrence, consent will be obtained for a further biopsy to assess how the tumour clonal structure has changed through therapy and disease progression. The primary objectives of TRACERx are to determine the relationship between intratumour heterogeneity and clinical outcome (disease-free survival [DFS] and overall survival [OS]), and to establish the impact of adjuvant platinum-containing regimens on intratumour heterogeneity in relapsed disease. The secondary objectives include developing and validating an intratumour heterogeneity index as a prognostic or predictive biomarker and identifying drivers of genomic instability, metastatic progression, and drug resistance by identifying and tracking the dynamics of somatic mutational heterogeneity. TRACERx also aims to define clonally dominant drivers of disease to address the role of clonal driver dominance in targeted therapeutic response, and to guide lung cancer treatment stratification. The sample collection per patient and overall study schema are summarised in Figure 1 and Figure 2, respectively.

## Spatial Heterogeneity and Branched Evolution in NSCLC

Previous efforts to characterise the cancer genome of NSCLC have involved the analysis of copy number alterations [11],[12], targeted sequencing of candidate cancer genes [13],[14] and next-generation sequencing of genomes and/or exomes [15]–[18]. By interrogating the mutational spectrum of tumours, these studies have demonstrated its complex and heterogeneous genomic landscape from point mutations to large structural variants, and the high mutational burden of smoking-related NSCLC. However, few studies in NSCLC have investigated the clonal and subclonal architecture of lung cancer tumours and their evolution through disease progression. The TRACERx consortium has developed methods to analyse the dynamics of genetic intratumour heterogeneity within individual tumours over time [7]. Distance-based phylogenetic trees will be inferred from the variants, insertions and deletions (INDELS), and structural variations observed in multiregion exome sequence datasets from a single tumour, allowing the discrimination of conserved early genetic mutations present at all sites of the primary tumour from later somatic events present in parts of the tumour and/or metastatic sites. This estimated temporal ordering will give insight into the potential relationships of such changes with ploidy shifts, chromosomal instability, and mutational processes that may change during the course of tumour progression.

## Histological Heterogeneity in NSCLC

Lung cancer is a histologically highly heterogeneous disease. Mixed lung tumours containing more than one histological type, such as adenosquamous tumours, combined small-cell tumours (small-cell combined with NSCLC), or tumours with areas of histological dedifferentiation are not uncommon. Within adenocarcinomas, histological variety is the rule, with most tumours showing a mixture of patterns, the commonest being lepidic/in situ, acinar, solid, papillary, and micropapillary. Solid and micropapillary patterns are associated with worse outcome [19]–[21]. Some patterns show associations with known driving mutations [22],[23], although these relationships are incompletely described at present. Furthermore, nuclear grade, which is not currently routinely assessed, often shows heterogeneity and is itself related to outcome [24],[25]. It is not known to what extent this spatial histological heterogeneity reflects genomic heterogeneity as opposed to epigenetic or microenvironmental influences. TRACERx aims to correlate histological heterogeneity with genomic heterogeneity and potentially improve the predictive and prognostic value of histological appearances in NSCLC.

## Tumour Heterogeneity, Outcome, and Impact of Platinum Chemotherapy in NSCLC

It is unclear why adjuvant chemotherapy following surgery for primary NSCLC is effective in some patients but not in others. An increasing body of evidence supports the association of patterns of intratumour heterogeneity, in multivariate analyses, with poor survival outcome in NSCLC and other solid tumours [26]. Indeed, work from us and others has shown that chromosomal instability, a driver of intratumour heterogeneity, is associated with cancer drug resistance, and numerous studies have documented the association of chromosomal instability with poor outcome in NSCLC [26]–[31]. The impact of intratumour heterogeneity on evolutionary fitness, together with the documented relationship of heterogeneity with drug resistance, supports the potential predictive nature of this candidate biomarker. Cytotoxic therapies have also been shown to influence the genomic landscape of drug-resistant diseases [32],[33], which raises the concern that increased genomic complexity in cytotoxic refractory tumours may potentiate tumour adaptation. However, studies to date are based on the analysis of small retrospective cohorts such that the true relationship between intratumour heterogeneity and clinical outcome, as well as the impact of platinum-based chemotherapy on the tumour genomic landscape, is currently unknown. TRACERx will prospectively assess whether an intratumour heterogeneity index can predict response to adjuvant therapy, and attempt to validate intratumour heterogeneity as an effective prognostic and predictive biomarker independent of known factors, such as tumour stage.

## Defining Drivers of Intratumour Heterogeneity and Drug Resistance

Deep sequencing analyses are revealing vast clonal heterogeneity present in solid tumours, including NSCLC, and the spatial and temporal dynamics of cancer subclones that emerge during the disease course and following acquired drug resistance [34],[35]. We have shown that drivers of intratumour heterogeneity can be defined in vivo and that one mechanism driving tumour heterogeneity in colorectal cancer, DNA replication stress, may be targetable [36]. Defining such processes in longitudinal solid tumour cohorts may have therapeutic relevance in attempting to limit tumour heterogeneity, adaptation, and cancer evolution [37]. TRACERx aims to develop an improved understanding of the relationship between phenotypic and genetic intratumour heterogeneity with cancer evolution, and identify further drivers of genomic instability. Ultimately it is hoped that this will support the development of novel therapeutic approaches to limit relapse and improve outcomes in NSCLC.

## Impact of Intratumour Heterogeneity on Host Immunity and Tumour Neo-Antigenic Repertoire

Whilst evidence suggests that intratumour heterogeneity may significantly limit the antitumour activity of targeted therapeutics [38], its overall effect on the anticancer immune response may be beneficial, since high levels of intratumoural mutational diversity may generate neo-antigens perceived by the immune system as non-self, thus providing relevant targets for immune-based therapies [38]–[40]. TRACERx aims to provide a resource to define the impact of intratumour heterogeneity on cancer immunity throughout tumour evolution and therapy. Through the integration of clinical and tumour multiregion sequencing data with immunological analysis, the consortium will assess various aspects of tumour immunobiology, including the overall impact of distinct drivers of intratumour heterogeneity on immune infiltration and function, the proportion of tumour infiltrating lymphocytes with the ability to recognise neo-antigens, and whether novel T cell receptors that recognise phosphopeptides preferentially expressed by tumour cells can be identified in patients, with NSCLC serving as a platform for the development of future immunotherapeutic strategies.

## Development of Minimally Invasive Methods to Study Tumour Evolution

Primary and metastatic tumours will be genetically profiled to identify clonal and subclonal driver mutations. However, our analysis of the primary tumour is limited to tissue surplus to diagnostic requirement, albeit multiregional, and our analysis of metastatic sites is likely to be restricted to one location, emphasising the need to develop less invasive approaches to follow tumour evolution. Circulating biomarkers have the potential to monitor minimal residual disease, forecast early progression, and document subclonal evolution through therapy and acquired drug resistance [41]. Here we propose to extend the TRACERx consortium's expertise in minimally invasive biomarker approaches to monitor tumour subclonal evolution through serial analysis of circulating-free tumour DNA (cfDNA) and circulating tumour cells (CTCs) before surgery and throughout the disease course. We have shown that cfDNA analysis is technically straightforward with limited cost and has demonstrable utility in disease monitoring [35],[42], and that CTC number in NSCLC is an independent prognostic biomarker [43]. TRACERx aims to address how intratumour heterogeneity is manifested in circulating biomarkers and the extent to which the genetic landscape of the primary and metastatic tumour is reflected in CTCs and cfDNA. This comparison will take into account the limitations in tumour sampling and therefore the potential to identify genetic aberrations in CTCs and cfDNA not detected in the tumour tissue. By comparing serial samples, TRACERx will determine whether cfDNA can detect residual disease following surgery and predict tumour recurrence, whether CTCs and cfDNA in advanced metastatic disease can represent further selection of subclones over time, and whether CTCs and cfDNA can provide insight into drug resistance mechanisms.

## Clonal Dominance and Clinical Outcome

There is a pressing need to define early driver events suitable for clinical drug trial stratification and to assess prospectively the role of drug target intratumour heterogeneity in the early emergence of resistance and poor DFS outcomes. Until recently, the term “actionable mutation” was used to define the presence of a somatic mutation or copy number event in a single tumour biopsy that might suggest a targeted therapeutic approach. However, emerging evidence for intratumour heterogeneity in breast cancer [44], renal cell carcinoma [7], glioblastoma [45], pancreatic cancer [6],[46], and medulloblastoma [47] demands the consideration of the role of clonal dominance when defining actionable events. In patients with an identified epidermal growth factor receptor (EGFR)-activating mutation treated with EGFR-targeted therapy, the clonality of the mutation is generally unknown, and yet it has been suggested that subclonal EGFR somatic mutational heterogeneity may be an understudied mechanism of drug resistance [48]. Similarly, identifying high-risk subclonal drivers that might contribute to metastatic progression and be suitable for therapeutic intervention is also an area of unmet need. Through the deep-sequencing of paired primary and relapsed-disease samples, TRACERx will distinguish clonal from subclonal drivers and will relate the clonal dominance of targetable events to progression-free survival (PFS) intervals for targeted therapies in the advanced disease setting within the DARWIN (Deciphering Anti-tumour Response With INtratumour Heterogeneity) Clinical Trial Programme that is currently in development. The consortium will attempt to define a new process for drug development, stratifying PFS outcomes based on clonal dominance of the targetable event, and map the tumour's subclonal dynamics during the acquisition of drug resistance.

## Metastatic Disease and Defining the Origins of the Lethal Subclone

Clonal diversity between primary and metastatic tumours in the same patient has been demonstrated in different tumour types, including but not limited to, breast [49], pancreatic [6],[46], prostate [9], and medulloblastoma [47]. Longitudinal sample collection and genomic analysis from the primary tumour through disease progression and at the time of death has the potential to identify the molecular features and subclonal origin of the metastatic process. In an interesting case of prostate cancer, Haffner and colleagues correlated whole-genome sequencing data from a primary tumour with three sites of metastases collected at autopsy 17 years after presentation [9]. Despite genetic heterogeneity among metastases, there were many shared events suggesting a monoclonal origin. Through histological assessment, alongside sequencing, they identified the lethal metastatic clone originating from the primary tumour. Patients who develop terminal metastatic NSCLC in TRACERx will be asked to consider enrolling in an autopsy programme that will be open nationally. For each patient, TRACERx will have accumulated an unprecedented amount of genetic data, and accessing tissue from multiple sites of disease after death would give some insight into the evolving constellation of genetic aberrations and a potential model for the metastatic process. Circulating biomarkers collected at this point may add to this model, although as previously mentioned, the extent to which these biomarkers reflect tumour genomics in NSCLC is yet to be fully determined.

## Conclusions

The importance of intratumour heterogeneity is increasingly recognised as a driver of tumour progression, drug resistance and treatment failure in solid tumours [5],[6],[27],[44],[47],[50]–[52]. The presence of subclonal driver events may prove a significant challenge to biomarker development and drug target discovery efforts, and contribute to drug resistance and poor survival outcome [10],[44],[53],[54]. Despite the impressive developments of international large-scale sequencing consortia, the spatial separation of tumour subclones, the changing nature of the disease over time, and the impact of such diversity upon outcome are yet to be addressed [3]. Lung TRACERx is a large-scale study integrating complex genomic data with phenotypic clinical annotation and outcome in order to decipher the heterogeneity of the cancer genome and mutational pathways involved in NSCLC pathogenesis. It aims to develop clinically meaningful measures of intratumour heterogeneity to guide patient management and treatment stratification [55] and to prospectively define thresholds of tumour heterogeneity for clinical risk stratification. With increasing awareness of the need to obtain tissue and genetically profile cancers in order to stratify treatment, the concept of longitudinal tissue collection and analysis has become more acceptable in oncological practice. In following cancers from diagnosis to relapse, tracking the evolutionary trajectories of tumours in relation to therapeutic interventions, and determining the impact of clonal heterogeneity on clinical outcomes, TRACERx may also serve as a model applicable to other cancer types.

TRACERx is not without its limitations. In determining the full extent of intratumour heterogeneity, we are reliant on tissue collected surplus to diagnostic requirements, and therefore entire tumours are not sequenced. However, with deep sequencing and multiregion sampling, together with retrospective genomics analysis of residual surplus tumour tissue guided by the metastatic sample datasets, we hope to achieve significant coverage of the relevant tumour genomic landscape within each patient. Analysing circulating biomarkers, such as cfDNA and CTCs, may further complement the tumour sequencing data and identify additional genetic aberrations not detected by primary or metastatic tumour sequencing. We anticipate that a biopsy of a metastatic site may not be appropriate in all patients, but having taken into account expected rates of attrition, we will have a sufficient number of cases to meet the study outcome objectives. Finally, our ability to detect subclonal somatic events occurring at low variant allele frequencies is limited by the power of our existing methods, but as sequencing and bioinformatics methods advance, TRACERx will adapt to incorporate such improvements in technologies, including the use of deep whole-genome sequencing datasets in some cases with no clear genetic driver events. Overall, TRACERx aims to develop an understanding of the genomic landscape of NSCLC through the disease course and the biological role of underlying genetic events that might contribute to disease progression. Optimising understanding of NSCLC evolutionary processes may help to identify novel therapeutic targets to improve clinical outcomes. As the cost of sequencing decreases and informatics techniques advance, such large-scale longitudinal genomic studies may become a central component to the delivery of precision cancer medicine.

## Abbreviations

cfDNA: circulating-free tumour DNA
CTC: circulating tumour cell
DFS: Disease-Free Survival
EGFR: epidermal growth factor receptor
GL DNA: germ line DNA
INDELS: insertions and deletions
NSCLC: Non-Small Cell Lung Cancer
OS: Overall Survival
PFS: Progression-Free Survival
Rx: therapy
TRACERx: TRAcking non-small cell lung Cancer Evolution through therapy (Rx)

## References

[1] Landau DA, Carter SL, Stojanov P, McKenna A, Stevenson K, et al (2013) Evolution and impact of subclonal mutations in chronic lymphocytic leukemia. Cell 152: 714–726. 23415222 10.1016/j.cell.2013.01.019 PMC3575604

[2] Stratton MR, Campbell PJ, Futreal PA (2009) The cancer genome. Nature 458: 719–724. 19360079 10.1038/nature07943 PMC2821689

[3] Yates LR, Campbell PJ (2012) Evolution of the cancer genome. Nat Rev Genet 13: 795–806. 23044827 10.1038/nrg3317 PMC3666082

[4] Nik-Zainal S, Van Loo P, Wedge DC, Alexandrov LB, Greenman CD, et al (2012) The life history of 21 breast cancers. Cell 149: 994–1007. 22608083 10.1016/j.cell.2012.04.023 PMC3428864

[5] Shah SP, Roth A, Goya R, Oloumi A, Ha G, et al (2012) The clonal and mutational evolution spectrum of primary triple-negative breast cancers. Nature 486: 395–399. 22495314 10.1038/nature10933 PMC3863681

[6] Campbell PJ, Yachida S, Mudie LJ, Stephens PJ, Pleasance ED, et al (2010) The patterns and dynamics of genomic instability in metastatic pancreatic cancer. Nature 467: 1109–1113. 20981101 10.1038/nature09460 PMC3137369

[7] Gerlinger M, Rowan AJ, Horswell S, Larkin J, Endesfelder D, et al (2012) Intratumor heterogeneity and branched evolution revealed by multiregion sequencing. N Engl J Med 366: 883–892. 22397650 10.1056/NEJMoa1113205 PMC4878653

[8] Thirlwell C, Will OC, Domingo E, Graham TA, McDonald SA, et al (2010) Clonality assessment and clonal ordering of individual neoplastic crypts shows polyclonality of colorectal adenomas. Gastroenterology 138: 1441–1454, 1454 e1441–1447. 20102718 10.1053/j.gastro.2010.01.033

[9] Haffner MC, Mosbruger T, Esopi DM, Fedor H, Heaphy CM, et al (2013) Tracking the clonal origin of lethal prostate cancer. J Clin Invest 123: 4918–4922. 24135135 10.1172/JCI70354 PMC3809798

[10] Anderson K, Lutz C, van Delft FW, Bateman CM, Guo Y, et al (2011) Genetic variegation of clonal architecture and propagating cells in leukaemia. Nature 469: 356–361. 21160474 10.1038/nature09650

[11] Weir BA, Woo MS, Getz G, Perner S, Ding L, et al (2007) Characterizing the cancer genome in lung adenocarcinoma. Nature 450: 893–898. 17982442 10.1038/nature06358 PMC2538683

[12] Tanaka H, Yanagisawa K, Shinjo K, Taguchi A, Maeno K, et al (2007) Lineage-specific dependency of lung adenocarcinomas on the lung development regulator TTF-1. Cancer Res 67: 6007–6011. 17616654 10.1158/0008-5472.CAN-06-4774

[13] Ding L, Getz G, Wheeler DA, Mardis ER, McLellan MD, et al (2008) Somatic mutations affect key pathways in lung adenocarcinoma. Nature 455: 1069–1075. 18948947 10.1038/nature07423 PMC2694412

[14] Kan Z, Jaiswal BS, Stinson J, Janakiraman V, Bhatt D, et al (2010) Diverse somatic mutation patterns and pathway alterations in human cancers. Nature 466: 869–873. 20668451 10.1038/nature09208

[15] Liu P, Morrison C, Wang L, Xiong D, Vedell P, et al (2012) Identification of somatic mutations in non-small cell lung carcinomas using whole-exome sequencing. Carcinogenesis 33: 1270–1276. 22510280 10.1093/carcin/bgs148 PMC3499051

[16] Lee W, Jiang Z, Liu J, Haverty PM, Guan Y, et al (2010) The mutation spectrum revealed by paired genome sequences from a lung cancer patient. Nature 465: 473–477. 20505728 10.1038/nature09004

[17] Govindan R, Ding L, Griffith M, Subramanian J, Dees ND, et al (2012) Genomic landscape of non-small cell lung cancer in smokers and never-smokers. Cell 150: 1121–1134. 22980976 10.1016/j.cell.2012.08.024 PMC3656590

[18] Imielinski M, Berger AH, Hammerman PS, Hernandez B, Pugh TJ, et al (2012) Mapping the hallmarks of lung adenocarcinoma with massively parallel sequencing. Cell 150: 1107–1120. 22980975 10.1016/j.cell.2012.08.029 PMC3557932

[19] Amin MB, Tamboli P, Merchant SH, Ordonez NG, Ro J, et al (2002) Micropapillary component in lung adenocarcinoma: a distinctive histologic feature with possible prognostic significance. Am J Surg Pathol 26: 358–364. 11859208 10.1097/00000478-200203000-00010

[20] Miyoshi T, Satoh Y, Okumura S, Nakagawa K, Shirakusa T, et al (2003) Early-stage lung adenocarcinomas with a micropapillary pattern, a distinct pathologic marker for a significantly poor prognosis. Am J Surg Pathol 27: 101–109. 12502932 10.1097/00000478-200301000-00011

[21] Barletta JA, Yeap BY, Chirieac LR (2010) Prognostic significance of grading in lung adenocarcinoma. Cancer 116: 659–669. 20014400 10.1002/cncr.24831 PMC2846761

[22] Ninomiya H, Hiramatsu M, Inamura K, Nomura K, Okui M, et al (2009) Correlation between morphology and EGFR mutations in lung adenocarcinomas Significance of the micropapillary pattern and the hobnail cell type. Lung Cancer 63: 235–240. 18571764 10.1016/j.lungcan.2008.04.017

[23] Shaw AT, Yeap BY, Mino-Kenudson M, Digumarthy SR, Costa DB, et al (2009) Clinical features and outcome of patients with non-small-cell lung cancer who harbor EML4-ALK. J Clin Oncol 27: 4247–4253. 19667264 10.1200/JCO.2009.22.6993 PMC2744268

[24] Kadota K, Suzuki K, Kachala SS, Zabor EC, Sima CS, et al (2012) A grading system combining architectural features and mitotic count predicts recurrence in stage I lung adenocarcinoma. Mod Pathol 25: 1117–1127. 22499226 10.1038/modpathol.2012.58 PMC4382749

[25] Petersen I, Kotb WF, Friedrich KH, Schluns K, Bocking A, et al (2009) Core classification of lung cancer: correlating nuclear size and mitoses with ploidy and clinicopathological parameters. Lung Cancer 65: 312–318. 19168259 10.1016/j.lungcan.2008.12.013

[26] McGranahan N, Burrell RA, Endesfelder D, Novelli MR, Swanton C (2012) Cancer chromosomal instability: therapeutic and diagnostic challenges. EMBO Rep 13: 528–538. 22595889 10.1038/embor.2012.61 PMC3367245

[27] Lee A, Endesfelder D, Rowan A, Walther A, Birkbak N, et al (2011) Chromosomal Instability Confers Intrinsic Multi-Drug Resistance. Cancer Res 71: 1858–1870. 21363922 10.1158/0008-5472.CAN-10-3604 PMC3059493

[28] Swanton C, Nicke B, Schuett M, Eklund AC, Ng C, et al (2009) Chromosomal instability determines taxane response. Proc Natl Acad Sci U S A 106: 8671–8676. 19458043 10.1073/pnas.0811835106 PMC2688979

[29] Kosaka T, Yatabe Y, Endoh H, Yoshida K, Hida T, et al (2006) Analysis of epidermal growth factor receptor gene mutation in patients with non-small cell lung cancer and acquired resistance to gefitinib. Clin Cancer Res 12: 5764–5769. 17020982 10.1158/1078-0432.CCR-06-0714

[30] Su KY, Chen HY, Li KC, Kuo ML, Yang JC, et al (2012) Pretreatment epidermal growth factor receptor (EGFR) T790M mutation predicts shorter EGFR tyrosine kinase inhibitor response duration in patients with non-small-cell lung cancer. J Clin Oncol 30: 433–440. 22215752 10.1200/JCO.2011.38.3224

[31] Turke AB, Zejnullahu K, Wu YL, Song Y, Dias-Santagata D, et al (2010) Preexistence and clonal selection of MET amplification in EGFR mutant NSCLC. Cancer Cell 17: 77–88. 20129249 10.1016/j.ccr.2009.11.022 PMC2980857

[32] Keats JJ, Chesi M, Egan JB, Garbitt VM, Palmer SE, et al (2012) Clonal competition with alternating dominance in multiple myeloma. Blood 120: 1067–1076. 22498740 10.1182/blood-2012-01-405985 PMC3412330

[33] Ding L, Ley TJ, Larson DE, Miller CA, Koboldt DC, et al (2012) Clonal evolution in relapsed acute myeloid leukaemia revealed by whole-genome sequencing. Nature 481: 506–510. 22237025 10.1038/nature10738 PMC3267864

[34] Swanton C (2012) Intratumor heterogeneity: evolution through space and time. Cancer Res 72: 4875–4882. 23002210 10.1158/0008-5472.CAN-12-2217 PMC3712191

[35] Diaz LA Jr, Williams RT, Wu J, Kinde I, Hecht JR, et al (2012) The molecular evolution of acquired resistance to targeted EGFR blockade in colorectal cancers. Nature 486: 537–540. 22722843 10.1038/nature11219 PMC3436069

[36] Burrell RA, McClelland SE, Endesfelder D, Groth P, Weller MC, et al (2013) Replication stress links structural and numerical cancer chromosomal instability. Nature 494: 492–496. 23446422 10.1038/nature11935 PMC4636055

[37] Burrell RA, McGranahan N, Bartek J, Swanton C (2013) The causes and consequences of genetic heterogeneity in cancer evolution. Nature 501: 338–345. 24048066 10.1038/nature12625

[38] Fisher R, Pusztai L, Swanton C (2013) Cancer heterogeneity: implications for targeted therapeutics. Br J Cancer 108: 479–485. 23299535 10.1038/bjc.2012.581 PMC3593543

[39] Peggs KS, Segal NH, Allison JP (2007) Targeting immunosupportive cancer therapies: accentuate the positive, eliminate the negative. Cancer Cell 12: 192–199. 17785201 10.1016/j.ccr.2007.08.023

[40] Segal NH, Parsons DW, Peggs KS, Velculescu V, Kinzler KW, et al (2008) Epitope landscape in breast and colorectal cancer. Cancer Res 68: 889–892. 18245491 10.1158/0008-5472.CAN-07-3095

[41] Aparicio S, Caldas C (2013) The implications of clonal genome evolution for cancer medicine. N Engl J Med 368: 842–851. 23445095 10.1056/NEJMra1204892

[42] Shaw JA, Page K, Blighe K, Hava N, Guttery D, et al (2012) Genomic analysis of circulating cell-free DNA infers breast cancer dormancy. Genome Res 22: 220–231. 21990379 10.1101/gr.123497.111 PMC3266030

[43] Krebs MG, Sloane R, Priest L, Lancashire L, Hou JM, et al (2011) Evaluation and prognostic significance of circulating tumor cells in patients with non-small-cell lung cancer. J Clin Oncol 29: 1556–1563. 21422424 10.1200/JCO.2010.28.7045

[44] Navin N, Kendall J, Troge J, Andrews P, Rodgers L, et al (2011) Tumour evolution inferred by single-cell sequencing. Nature 472: 90–94. 21399628 10.1038/nature09807 PMC4504184

[45] Sottoriva A, Spiteri I, Piccirillo SG, Touloumis A, Collins VP, et al (2013) Intratumor heterogeneity in human glioblastoma reflects cancer evolutionary dynamics. Proc Natl Acad Sci U S A 110: 4009–4014. 23412337 10.1073/pnas.1219747110 PMC3593922

[46] Yachida S, Jones S, Bozic I, Antal T, Leary R, et al (2010) Distant metastasis occurs late during the genetic evolution of pancreatic cancer. Nature 467: 1114–1117. 20981102 10.1038/nature09515 PMC3148940

[47] Wu X, Northcott PA, Dubuc A, Dupuy AJ, Shih DJ, et al (2012) Clonal selection drives genetic divergence of metastatic medulloblastoma. Nature 482: 529–533. 22343890 10.1038/nature10825 PMC3288636

[48] Chen ZY, Zhong WZ, Zhang XC, Su J, Yang XN, et al (2012) EGFR mutation heterogeneity and the mixed response to EGFR tyrosine kinase inhibitors of lung adenocarcinomas. Oncologist 17: 978–985. 22673630 10.1634/theoncologist.2011-0385 PMC3399655

[49] Shah SP, Morin RD, Khattra J, Prentice L, Pugh T, et al (2009) Mutational evolution in a lobular breast tumour profiled at single nucleotide resolution. Nature 461: 809–813. 19812674 10.1038/nature08489

[50] Szerlip NJ, Pedraza A, Chakravarty D, Azim M, McGuire J, et al (2012) Intratumoral heterogeneity of receptor tyrosine kinases EGFR and PDGFRA amplification in glioblastoma defines subpopulations with distinct growth factor response. Proc Natl Acad Sci U S A 109: 3041–3046. 22323597 10.1073/pnas.1114033109 PMC3286976

[51] Xu X, Hou Y, Yin X, Bao L, Tang A, et al (2012) Single-cell exome sequencing reveals single-nucleotide mutation characteristics of a kidney tumor. Cell 148: 886–895. 22385958 10.1016/j.cell.2012.02.025 PMC7458411

[52] Yap T, Gerlinger M, Futreal A, Pustzai L, Swanton C (2012) Intratumour Heterogeneity: Seeing the wood for the trees. Sci Transl Med 4: 127ps10. 10.1126/scitranslmed.3003854 22461637

[53] Swanton C, Burrell RA, Futreal PA (2011) Breast cancer genome heterogeneity: a challenge to personalised medicine? Breast Cancer Res 13: 104. 21345264 10.1186/bcr2807 PMC3109569

[54] Navin N, Krasnitz A, Rodgers L, Cook K, Meth J, et al (2010) Inferring tumor progression from genomic heterogeneity. Genome Res 20: 68–80. 19903760 10.1101/gr.099622.109 PMC2798832

[55] Merlo LM, Shah NA, Li X, Blount PL, Vaughan TL, et al (2010) A comprehensive survey of clonal diversity measures in Barrett's esophagus as biomarkers of progression to esophageal adenocarcinoma. Cancer Prev Res (Phila) 3: 1388–1397. 20947487 10.1158/1940-6207.CAPR-10-0108 PMC3004782

